# Noise and sound in the intensive care unit: a cohort study

**DOI:** 10.1038/s41598-025-94365-8

**Published:** 2025-03-29

**Authors:** A. Tahvili, A. Waite, T. Hampton, I. Welters, P. J. Lee

**Affiliations:** 1 Institute for Life Course and Medical Sciences, University of Liverpool, Liverpool, UK; 2 Intensive Care Unit, Royal Liverpool University Hospital Liverpool University Hospitals NHS Foundation Trust, Liverpool, UK; 3 Department of Clinical Sciences, Liverpool School of Tropical Medicine, Pembroke Pl, Liverpool, Merseyside L3 5QA UK; 4 Acoustics Research Unit, School of Architecture, University of Liverpool, Liverpool, UK

**Keywords:** Noise, Intensive care units (ICU), Acoustics, Health facilities, Occupational health, Health care, Acoustics

## Abstract

Intensive care units (ICUs) are acknowledged for their propensity for noise, often exhibiting higher sound levels on average than other departments. This is mainly ascribed to a high concentration of medical devices and staff, creating an acoustic environment characterised by a high level of staff activity and a concoction of alarms from therapeutic and monitoring devices. Excessive noise in ICUs has been associated with adverse health effects and human factor impacts acknowledged to negatively affect both patients and healthcare providers. This study aimed to evaluate the sound levels of the Royal Liverpool University Hospital (RLUH) ICU and compare it against recommended guidelines. Prospective sound level measurements were taken from a six-bedded bay within the RLUH ICU between 15th June and 1st July 2022. This audit focussed on sound data that equal or exceeded 87 dBA, in accordance with levels in the UK Noise Regulations. The data involved 11 patients admitted to the bay within the defined timeframe. A retrospective review of the patients’ records was conducted to identify potential noisy events during the recording period. Results revealed all L_Aeq_ and *L*_Amax_ measurements exceeded the recommended guidelines. Although HSE exposure limit values were not exceeded, the lowest *L*_Amin_ value recorded was 44.2 dBA and only one hour from 16 days of recording (less than 1% of the time) fell below international daytime guidelines of 45 dBA. The top documented potential causes of noise were patient repositioning/personal care, medication administration and suctioning. Sound levels in the RLUH ICU considerably exceed national and international guidelines. These findings highlight the need to address the issue of noise pollution in the ICU setting. Hospital staff should consider implementing strategies and interventions for noise reduction in ICUs.

## Introduction

Patients and healthcare staff are consistently exposed to high levels of noise in hospital^1^. Since the 1960s, there has been a rise in average daytime and night-time sound levels^2^, which is largely attributed to the increased use of advanced medical technology^3^. Intensive care units (ICUs) are acknowledged for their propensity for noise, often exhibiting higher average sound levels than other areas within hospital^3,4^. This is mainly ascribed to a high concentration of medical devices and staff in ICUs, creating a soundscape characterised by a high level of staff activity, background noise, and an acoustic concoction of alarms from therapeutic and monitoring devices^3,5^.

An increasing body of research on noise pollution has reported associations with short- and long-term adverse effects on health^2,6^. Previous simulation studies have suggested that medical device alarms alone may not be the source of noise and that critical care itself may be noisy^1^. Excessive ambient sound levels in the ICU have been acknowledged as a major contributor to sleep disturbance among patients^7–9^. Noise can elicit changes in sleep depth, disrupting the sleep-wake cycle and causing a subjective deterioration in sleep quality^10,11^. A literature review conducted by Xie et al. suggested that noise may account for up to 76% of sleep disturbance in ICU patients^12^. However, the key studies included in their review were heterogenous with small sample sizes^12^. During sleep, excessive noise exposure triggers the inflammatory response and disrupts endothelial function, resulting in oxidative stress that can adversely affect the vasculature of vital organs and may ultimately contribute to the development of various clinical conditions^13^. Several studies have demonstrated the potential wide-ranging adverse effects of sleep deprivation, encompassing psychological disturbances^14^, immune system disruption^15^, an increased incidence of cardiovascular disease^16^ and respiratory deterioration^17,18^, including apnoea periods and challenges in weaning patients from ventilatory support^19^. Importantly, a pertinent consequence in ICU patients is the development of delirium^20^. Whilst in ICU, between 30% and 75% of patients experience delirium^21^. Patients who experience delirium have prolonged hospital admissions, higher morbidity and mortality, and may experience ongoing cognitive impairment after discharge^21^. In a recent meta-analysis the use of earplugs significantly decreased the risk of delirium in ICU patients^22^. Similarly, Van de Pol et al. found that implementing a noise reduction protocol, which included the use of earplugs, significantly reduced the incidence of delirium among ICU patients^23^.

In addition to its impact on patients, high noise levels in health settings can have adverse effects on healthcare staff^24^. Noise is the most commonly encountered performance obstacle among critical care nurses^25^, can negatively impact work performance^26^, and increases the likelihood of medical errors^27^. Empirical evidence has demonstrated a strong correlation between excessive noise and heightened levels of fatigue, irritation, and stress among nurses operating in ICUs^28–31^, with excessive noise emerging as a contributing risk factor for occupational burnout^28,32^. Moreover, alarm fatigue and burnout experienced by critical care staff have the potential to reduce work performance and negatively affect patient outcomes^33,34^. Song et al. reported that voice strain among ICU nurses correlated with excessive workplace noise^35^. Beyond the direct implications for staff well-being, research has also raised patient-safety concerns, given that excessive noise has also been shown to lead to miscommunication between staff and hinder concentration when performing tasks^36–39^. The cognitive expense of subconscious processing of distracting noise restricts the brain’s capacity to process visual and auditory information^40^. Self-assessment surveys conducted among ICU staff found significantly higher distraction ratings, higher stress levels, and reduced confidence in performance following noise exposure^41^.

The World Health Organisation (WHO) suggest A-weighted sound level (dBA) of approximately 50 to 55 dBA during daytime hours and 40 to 45 dBA overnight is acceptable to the average healthy adult^6^, with some variance in comfort according to individual sensitivity to noise^42^. Within this range, the majority of individuals would not experience significant adverse health effects or sleep disruption^6^. However, quantifiable effects of noise on sleep have been observed at equivalent sound pressure levels (*L*_Aeq_) as low as 30 dBA, with a concomitant peak sound level (*L*_Amax_) threshold of 45 dBA^6^. Table 1 provides a detailed outline of the noise measurement parameters, including dBA and *L*_Aeq_.

**Table 1:** Noise measurement parameters.

Term	Description
Decibel, dB	A logarithmic unit used to measure sound pressure level^43^
A-weighted sound level in decibels, dBA	A unit of measurement that adjusts sound levels to the sensitivity of the human ear to different frequencies. dBA represents the relative loudness of sound, as perceived by the human ear^43^
Equivalent continuous sound level, *L*_Aeq_	The average sound pressure over an indicated time interval^43^
Maximum sound level, *L*_Amax_	The maximum time-weighted sound level (*L*_Aeq_) measured during a time interval^43^

The WHO^6^, International Noise Council (INC)^44^, and the United States Environmental Protection Agency (USEPA)^45^ have all published guidelines pertaining to sound pressure levels in hospitals. Among them, the WHO have set the most stringent threshold standard, with a recommended limit of 35 dBA during the day and 30 dBA at night^6^. By contrast, the guidelines provided by the INC and USEPA propose slightly higher thresholds^44,45^. Table 2 provides an overview of the standard *L*_Aeq_ set by each organisation.

**Table 2:** The recommended set equivalent continuous sound pressure level (*L*_Aeq_) for hospitals as defined by the separate organisations.

	WHO,^6^ dBA	International noise council,^44^ dBA	USEPA,^45^ dBA
Day	35	45	45
Evening	–	40	–
Night	30	20	35

*WHO* World health organisation, *USEPA* United States environmental protection agency, *dBA* A-weighted sound level.

Previous research has revealed a consistent upward trend in the already elevated noise levels within ICUs, with an annual increment of 0.38 dBA during the day and 0.42 dBA at night^3^. Notably, the average diurnal sound levels rose from 57 dBA in 1960 to 72 dBA in 2005, while nocturnal levels increased from 42 dBA to 60 dBA over the same time period^3^. Consequently, sound levels observed in the ICU persistently surpass the recommended thresholds stipulated by the WHO^46–49^. Research conducted in daily clinical practice has shown that average sound levels in the ICU range between 51 dBA to 70 dBA^3,50–52^, which is comparable to the noise levels encountered in heavy traffic^6^. Additionally, studies have observed peak sound levels in the ICU exceeding 80 dBA^49,53,54^, generally attributable to the operation of IV infusion pumps, monitor alarms, and ventilators^53^. A UK study conducted across five adult ICUs demonstrated peak sound levels above 100 dBA occurring 22–28 times per hour^52^. Peaks exceeding 85 dBA were observed at all sites, up to 16 per hour at night and more frequently throughout the day^52^. Considering these effects of noise pollution in the ICU for patients and health care staff, an analysis of the sources of high noise levels could allow for increased attention and mitigating strategies.

### Aims

The primary aim of this study is to comprehensively assess the sound levels in patient care areas within the ICU at royal liverpool university hospital (RLUH) and ascertain whether the measured sound levels comply with current standards and guidelines. As a secondary outcome, this study aims to evaluate the circumstances in which guidelines are breached.

## Methods

This retrospective, observational study was designed to evaluate the noise levels and their potential sources on the ICU department at the RLUH. The study followed an approved audit protocol registered with the Liverpool University Hospitals Foundation Trust (LUHFT) Audit Department (project 11338). The hospital audit and research department determined that additional ethical approval was not required as this was an ongoing service improvement project.

### Sound level measurements

Prospective continuous sound pressure level measurements were taken in one location within a six-bedded bay of patients. Maximum, mean and minimum sound pressure levels were obtained for each one-minute period between the start of recording at 12:52 on 15th June 2022 and 18:25 on 1st July 2022, providing 23,374 min of sound pressure data. Measurements were taken using a Class 1 integrating sound level meter (Brüel & Kjær, Type 2250) calibrated to 94 dB at 1 kHz both before and after the measurements using a sound calibrator (Brüel & Kjær, Type 4230).

The sound level meter was located centrally within the open bay, adjacent to the nurses station but outside any one patient’s bed space and the microphone was positioned 0.5 m above the level of the average patient’s head. It was also placed at least 1 m away from hard surfaces.

As all sound levels were above those recommended by the World Health Organisation (WHO), USEPA and International Noise Council (see Table 2), all the potential causes of noise during the study period were assessed by retrospective review of the patients’ medical records, against pre-defined parameters using a REDCap-based electronic form^55^.

Patients included in the study were admitted to the designated bay at different stages in their overall ICU admission, ranging from their first to the 84th day. All patients had at least one form of organ support during their stay. Five patients received respiratory support only (45.5%). Among the five patients who required two forms of organ support, four patients received cardiovascular and respiratory support and one patient received renal replacement therapy and respiratory support. Only one patient needed support for three organs. We additionally assessed all 1-minute timepoints where sound pressure ≥ 87 dBA. These specific data were deliberately selected since they surpass the figure used in the national exposure limit value (over 8 h) set by the Health and Safety Executive (HSE) which aims to mitigate health and safety risks associated with noise exposure in occupational settings^56^. The exposure limit value represents the maximum permissible peak sound pressure that must not be exceeded for any employee according to HSE guidelines^56^. Although we investigated single time point sound when LAmax ≥ 87 dBA, this did not necessarily correlate with exceeded exposure, and for reassurance in the results, the formula below was used to calculate the actual daily exposure level (LEP) for comparison with the HSE Noise in Work (2005) regulations (upper exposure action values: daily or weekly exposure of 87 dBA, lower exposure value of 80 dBA)^56^.

$${\text{LEP}}\,=\,{L_{{\text{Aeq}},{\text{T}}}}+{\text{1}}0*\left( {{\text{Te}}/{\text{To}}} \right)$$

Te: the duration of the workday in seconds.

T0: 28,8000 s, representing a standard 8-hour workday.

e.g. If the *L*_Aeq_ is 55 dBA for 8 h, then the LEP is 55 dBA.

### Data collection

To identify potential instances of noisy events during their stay, a retrospective review of the patients’ medical records was conducted by the first author. This review involved thorough examination of both handwritten ICU observation charts and Patient Electronic Notes System (PENS), the in-house electronic document management system utilised at the RLUH. For the purpose of data collection, a proforma was developed using research electronic data capture (REDCap) tools hosted at the University of Liverpool^55^. The synthesis of REDCap forms was informed by existing literature, ensuring their alignment to established evidence^9,36,52,57,58^. REDCap form A and B (see appendix) collected patient demographic data, and itemised ‘daily’ data respectively. Detailed inclusion and exclusion criteria are provided in Table 3 (also in appendix).

**Table 3:** Inclusion and exclusion criteria for data collection.

Inclusion	Exclusion
Sound measurements taken from one location within a six-bedded bay of patients in the RLUH ICU between 15/06/2022 12:52 and 01/07/2022 18:25	Other speciality areas
	Staff only or corridor areas, where patient care is not provided
Data collection of patient records within the specified 16 day audit period.	Data outside the specified audit period
Patient records sourced from both PENS and handwritten ICU observation charts	Patient records that are not available in either PENS or ICU observation charts

### Data analysis

Data is reported using descriptive statistics. The software used for data analysis was Microsoft Excel, (2019) (Microsoft Corporation, USA).

## Results

### Patient demographic

Within the defined study period, there were a total of 11 patients admitted to the ICU bay. Patient demographic data for this study is summarised in (Table 4).

**Table 4:** Patient demographic data, APACHE II score, length of admission and organ support requirement APACHE II acute physiology and chronic health evaluation II^59^.

Patient characteristics (total *n* = 11)	
Age (years), median [IQR]	64 [29]
Male sex, n	5
Female sex, n	6
APACHE II score, median [IQR]	16 [5]
Duration of ICU admission in days, median [IQR]	13 [11.5]
Number of organs supported during ICU admission, n (%)	
1	5 (45.5)
2	5 (45.5)
3	1 (9.0)

Patients included in the study were admitted to the designated bay at different stages in their overall ICU admission, ranging from their first to the 84th day. All patients had at least one form of organ support during their stay. Five patients received respiratory support only (45.5%). Among the five patients who required two forms of organ support, four patients received cardiovascular and respiratory support and one patient received renal replacement therapy and respiratory support. Only one patient needed support for three organs.

### Sound pressure level measurements

**Fig. 1:**
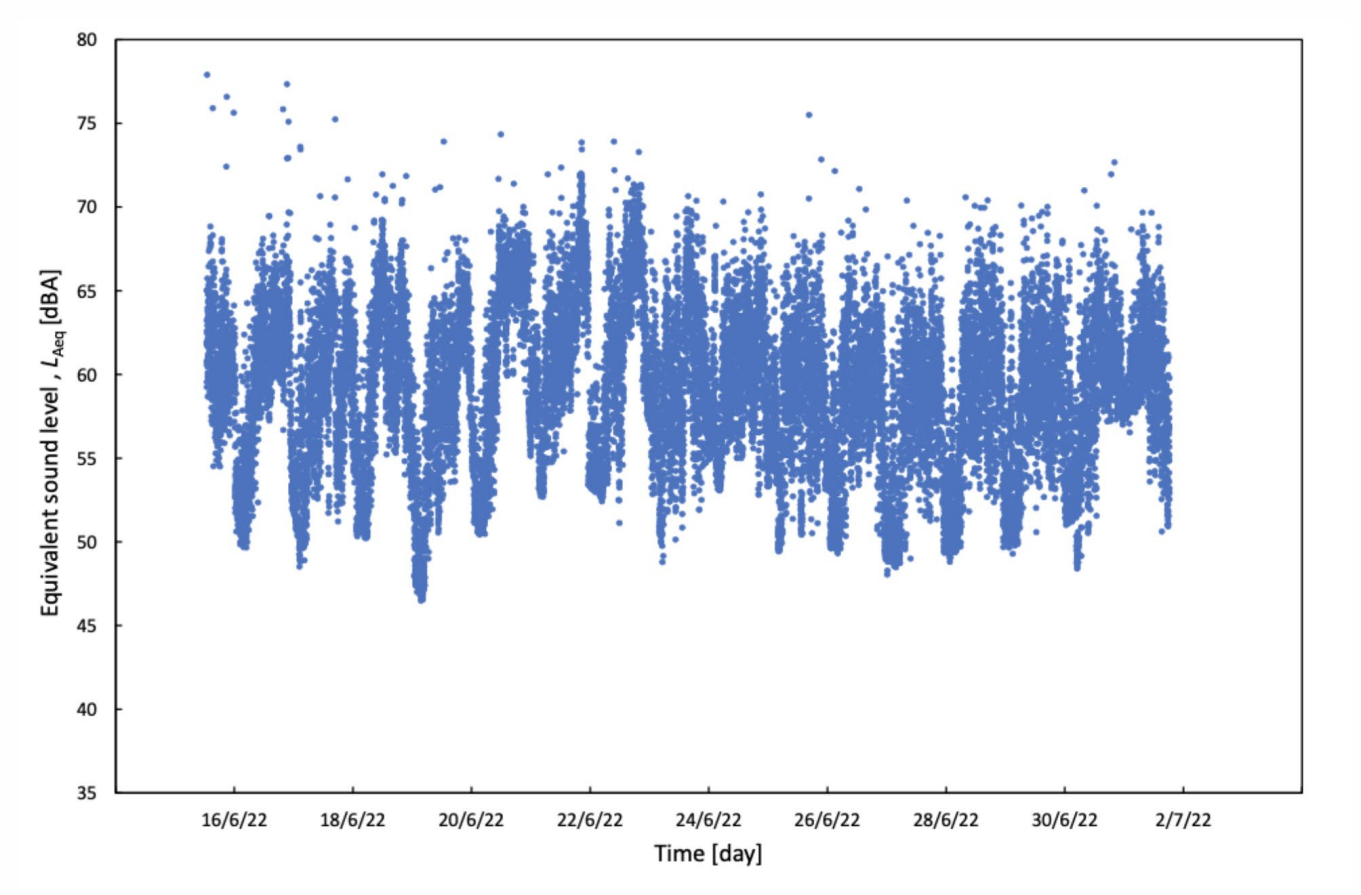
Scatter graph showing the *L*_Aeq_ values corresponding to each 1-minute recording obtained in the study period. *L*_Aeq_ equivalent continuous sound level, *dBA* A-weighted sound level.

**Fig. 2:**
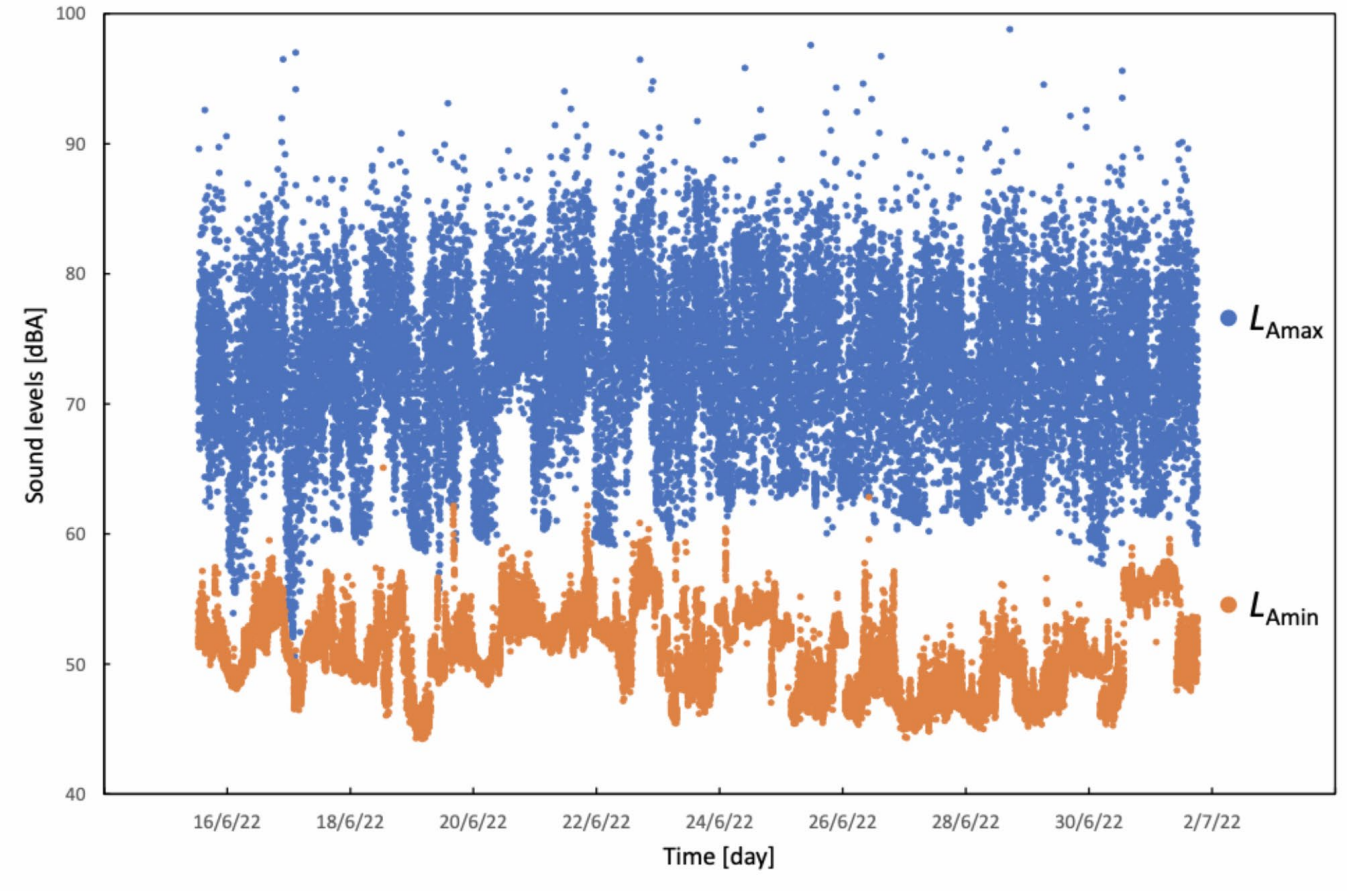
Scatter graph showing both *L*_AFmax_ and *L*_AFmin_ values corresponding to each 1-minute recording obtained in the study period. *L*_Amax_ maximum sound level, *L*_Amin_ minimum sound level, *dBA* A-weighted sound level.

The comprehensive dataset of all the sound level data is depicted in (Figs. 1 and 2). The lowest *L*_Aeq_ over 1 min recorded among all measurements was 46.7 dBA. The corresponding *L*_Amax_ and *L*_Amin_ for this recording weres 59.3 and 44.7 dBA, respectively. Additionally, the lowest *L*_Amax_ recorded in a single minute among all the measurements was 50.6 dBA, with corresponding *L*_Aeq_ and *L*_Amin_ values of 48.5 and 47.5 dBA, respectively. The lowest *L*_Amin_ value over a minute recorded amongst the measurements was 44.2 dBA. Notably, only a minority of *L*_Amin_ values (*n* = 60, 0.36%, or only 1 h from 16 days) fell below the daytime guideline of 45 dBA set by INC and USEPA.

**Fig. 3:**
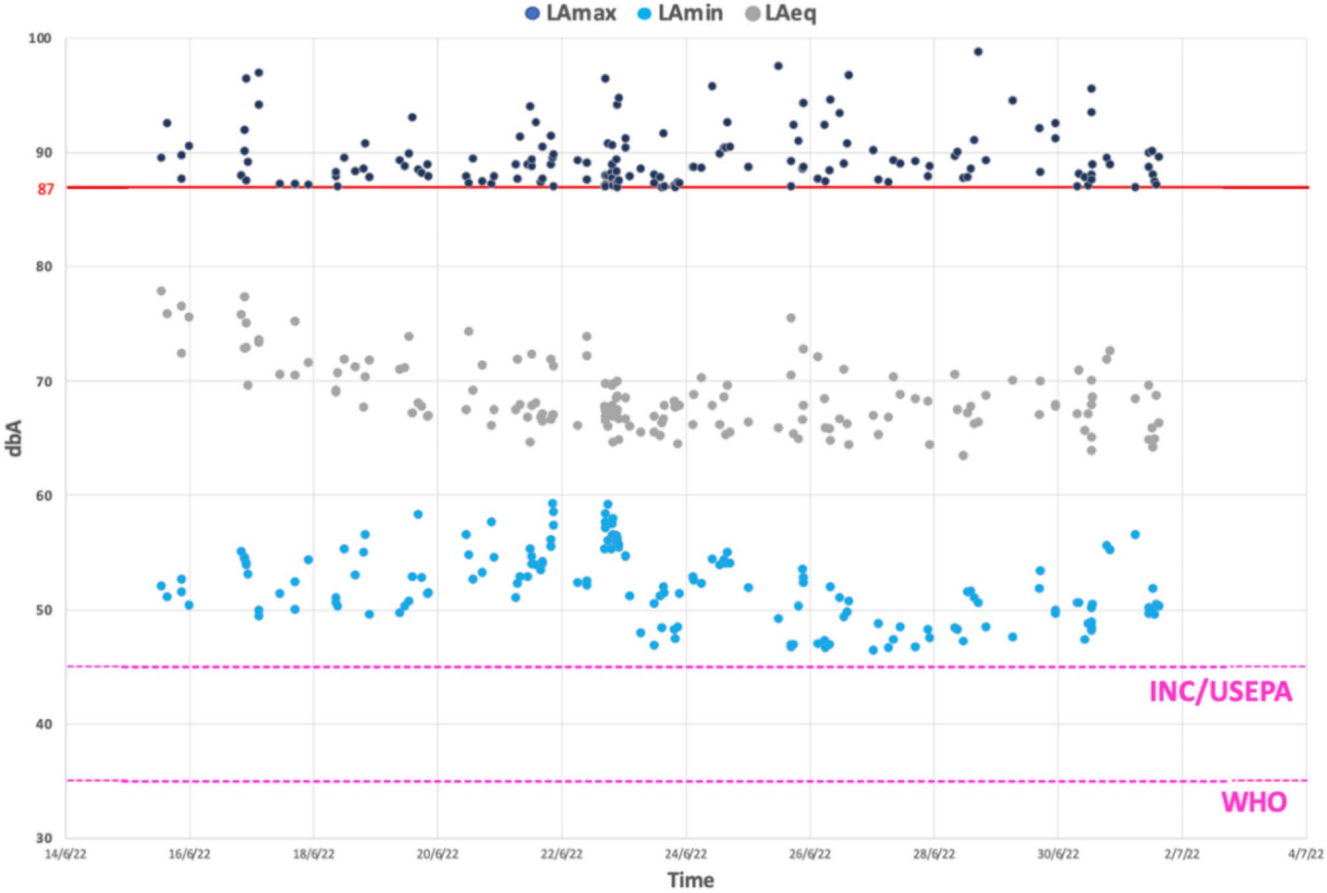
Scatter graph showing the loudest timepoints (*L*_Amax_ ≥87.0 dBA) with reference lines to show the noise exposure limit recommended threshold daytime *L*_Aeq_ set by the World Health Organisation (WHO),^6^ international noise council (INC)^43^ and the United States Environmental Protection Agency (USEPA)^44^. The red line represents an *L*_Amax_ of 87 dBA but this does not represent the HSE daily personal exposure level (LEP). See results below for further explanation. *L*_Amax_ maximum sound level, *L*_Amin_ minimum sound level, *L*_Aeq_ equivalent continuous sound level, *dBA* A-weighted sound level.

Figure 3 presents the sound level data of primary interest in this study. This subgroup analysis of the main dataset consists of 168 1-minute recordings where the *L*_Amax_ value ≥ 87.0 dBA. The highest *L*_Amax_ and *L*_Amin_ values recorded within this subgroup were 98.8 dBA and 59.3 dBA respectively. The lowest *L*_Aeq_ value recorded in this subgroup was 63.5 dBA. Conversely, the highest *L*_Aeq_ value observed in this subgroup was 77.9 dBA, with corresponding *L*_Amax_ and *L*_Amin_ values of 89.6 dBA and 52.1 dBA, respectively. *L*_Aeq_ values exceeded 65.0 dBA for most timepoints (*n* = 148, 88%). The mean *L*_Aeq_ from this data was calculated to be 68.6 dBA.

### Potential noisy events in ICU

**Fig. 4:**
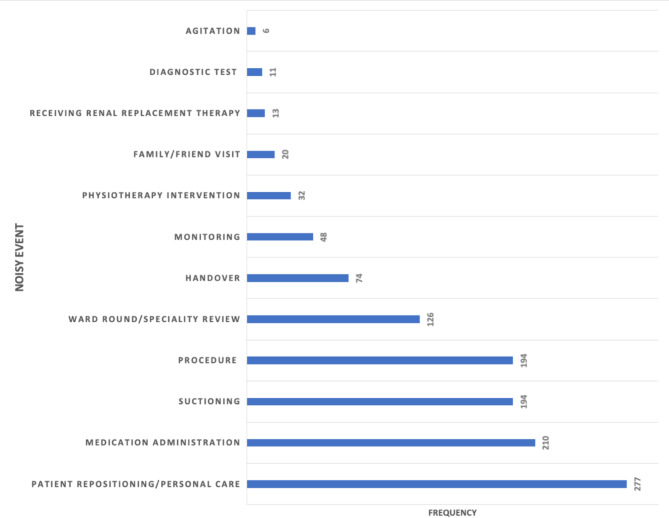
Cumulative frequency of documented potential noisy events.

Among the loudest timepoints (exceeding 87 dBA), multiple sources of noise were identified for most of these louder periods. The most common potential causes of noise were patient repositioning/personal care (identified 277 times across the whole recording time), medication administration (210 times), suctioning (194 times) and procedures (194 times), as shown in (Fig. 4). Notably, most of these procedures involved taking bloods (*n* = 163) (which can occur during line insertions, routine reviews or a deterioration in patient condition). A noisy subset of procedures involved interventions such as extubation (*n* = 3), tracheostomy insertion (*n* = 2) and a singular occurrence of ascitic drain removal.

**Fig. 5:**
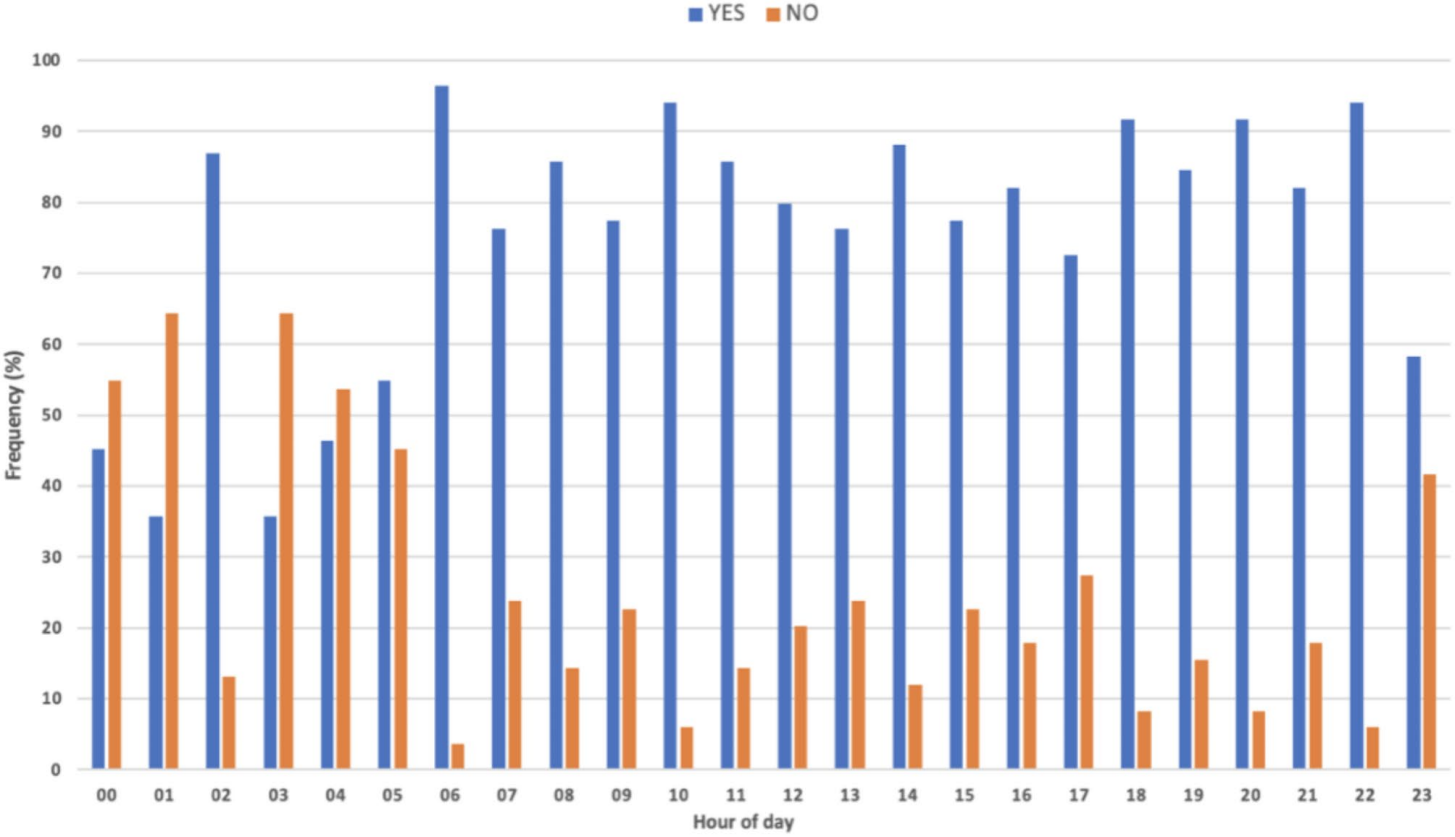
Bar chart showing the percentage frequency of hourly presence (‘Yes’) or absence (‘No’) of potential noisy events in the RLUH ICU. YES = presence of ≥ 1 potential noisy event in patient records, NO = no potential noisy event identified within patient records.

Figure 5 shows the frequency of the presence or absence of documented, potentially noisy events per hour for all patients within the bay during the defined study period. The frequency of at least one potential noisy event being present exceeded 70% for 18 out of 24 h. During the hours of 23:00 to 02:00 and 03:00 to 06:00, there was a comparatively lower frequency of noisy events present per hour versus daytime hours (06:00 to 21:00). The hours with the noisiest events were 6am, 10am, 6pm, 8pm and 10pm. By contrast, the hour of 1am exhibited the least noisy events (*n* = 30, 35.7%). Across the study period, there were noisy events in 96.4% of minutes between 6am and 7am. By contrast the hour of 1am exhibited the least noisy events (*n* = 30, 35.7%).

### Cumulative prevalence of noisy events in the RLUH ICU

The aforementioned frequently documented potential causes of noise were patient repositioning/personal care, medication administration, procedures and suctioning. Notable rises in the frequency of patient repositioning/personal care were observed in the hours of 2am (*n* = 68), 6am (*n* = 67), and 10pm (*n* = 65). Similarly, surges in frequency of medication administration occurred at the hours of 6am (*n* = 54), 6pm (*n* = 46) and 10pm (*n* = 42). Monitoring abnormalities were observed throughout each hour with a peak seen at 2pm (*n* = 11). These abnormalities primarily involved patients’ blood pressure (*n* = 21) followed by heart rate (*n* = 15) and saturations (*n* = 15).

### Cumulative personal exposure to noise (HSE guidelines)

Table 5 shows the converted *L*_Aeq_ values to daily personal exposure level (LEP) for the patients in this study across all 14 days of the recording. The highest LEP from the measurement is 69.0 dBA.

**Table 5:** Daily personal exposure level (LEP) for study duration in LE_P_ dB A.

	Day of study													
LE_P_ dB A	1	2	3	4	5	6	7	8	9	10	11	12	13	14
24 h	66.2	65.3	66.3	65.1	68.7	68.3	69.0	66.4	66.0	65.0	64.8	63.8	65.4	65.2
Day	64.0	63.2	64.9	62.0	66.6	64.9	66.9	64.4	63.6	63.0	63.1	62.0	63.8	63.1
Evening	60.8	59.0	59.7	60.9	63.1	65.0	63.5	58.7	60.4	58.5	57.7	57.1	58.4	58.6
Night	56.8	57.3	53.9	56.3	59.4	57.0	59.3	59.4	57.9	56.5	55.6	54.7	56.1	57.2

## Discussion

### Sound levels

The results of this audit reveal that sound levels in the RLUH Intensive Care Unit considerably exceeded the recommended guidelines set by the WHO^6^, INC^44^ and USEPA^45^. Patients in the RLUH ICU were continually exposed to a sound level which, at its quietest, was approximately just below conversation level^60^ and more often comparable to the sound produced by a nearby television^60^. No *L*_Aeq_ value recorded among the total sound data was compliant with the 35 dBA parameter set by the WHO^6^.

The findings of this study align with previous research conducted in ICU^49,52,61–63^. The highest *L*_Amax_ in this study reached 98.8 dBA, which is almost 10 dBA higher than the peak *L*_Amax_ level observed in a Swedish neurosurgical ICU^62^ and a Turkish surgical ICU^61^. 88% of *L*_Aeq_ values in this dataset exceeded 65 dBA, contrasting a study conducted across the Thames Valley, United Kingdom, which involved five ICUs, including four adult ICUs and one neurosurgical ICU, and reported their maximum *L*_Aeq_ as 59.9 dBA^52^. The observed differences highlight the presence of heterogeneity among ICUs, one possible contributing factor could be variations in specialty care among hospitals, encompassing patient demographics, architectural design, staffing levels, and visiting time restrictions. The *L*_Amax_ values recorded in this study are all in excess of the WHO guideline of 40 dBA overnight^6^, by a margin of at least 10 dBA. A 10 dBA increase in sound level corresponds to a doubled sound intensity perceptible to the human ear^60^. These findings are consistent with observations reported by MacKenzie et al. in their study across two ICUs in Edinburgh^46^.

The HSE guideline aims to provide hearing protection for workers exposed to extremely high noise levels, such as machinery noise, so the limit value (86 dBA) is set to be very high. Clinicians have anecdotally reported misinterpreting this 86 dBA figure as *L*_Aeq_ or *L*_Amax_ equivalent, so our findings (highest LEp 69 dBA) may be able to reasuure non-acousticians auditing their own units, that even when sound levels exceed WHO guidance, they fall far below the HSE action level.

### Sound sources in the ICU

Previous literature consistently identifies alarms, clinical equipment^38,64,65^ and staff communication^9,38,46,49,66^ as prominent sources of noise in ICUs. In this study, patient repositioning/personal care, medication administration and suctioning emerged as the most frequently documented potential causes of noise. In their comprehensive analysis of noise sources in acute care units including ICUs within Edinburgh hospitals, MacKenzie et al.^46^. reported that while patient repositioning and care can cause bed rail clinking that produces sound levels in the 70 to 80 dBA range, the occurrence rate of this noise source was relatively low, accounting for only 79 occurrences time across their 24-hour study period^46^. By contrast, they identified talking between staff as one of the top sources of noise, with 486 occurrences over the same timeframe^46^. Although not specifically cited as an avoidable noise source in their research^46^, existing literature suggests that staff education can contribute to the reduction of noise levels^65,67^. For instance, the use of ‘quiet’ signs outside patient-care areas^67^ and dimming the light level in wards have shown to lead to quieter conversations among staff^65^. Similarly, Tegnestedt et al. reported 64% of disruptive sounds were caused by monitoring alarms and staff conversations not relating to patient care in their observational study of three rooms in an ICU^68^. Song et al. found that the most common noise sources in four Chinese ICUs were talking and footsteps^49^. Moreover, they observed that the sound exposure level and maximum sound levels from voices and talking were greater than those from other sources^49^. However, they did not discuss the specific clinical activities linked to these noise sources.

Darbyshire et al. conducted a study mapping sources of noise in an adult ICU and found that a substantial portion of loud noise originated at the bedside, primarily from physiological monitors and ventilators positioned near patients’ ears^36^. These devices generally produced minimal sound, except when alarms were activated and emitted noise levels exceeding 50 dBA^36^, when the frequency range was comparable to that of a human scream^36^. They noted that although staff had the ability to adjust the volume settings of monitors and ventilators, they rarely modified them from their default setting^36^.

Although our study did not identify monitoring abnormalities as the commonest potential source of noise, research has shown that a majority of monitoring alarms (85 to 99.5%)^69–71^ are clinically irrelevant, and this is mainly attributed to maladjusted vital parameter alarms leading to a high incidence of ‘false positives’ (90%)^72^. Therefore monitoring alarms are often perceived as unhelpful by medical staff^73^. Clinicians may become accustomed to the acoustic environment, such as loud noise sources and reverberations of their individual ICU and this may lead to a selective disregard for what is perceived as the background noise of care, including regular low-level alarming or noise^74^. This phenomenon may occur even if such noise has clinical significance^75^. As a result, alarm fatigue can contribute to a delayed response by clinicians, posing a potential threat to patient safety^76^. Reduction of false alarms can be achieved through adopting better alarm management strategies, such as personalising the selection of monitoring elements for each patient and implementing patient-specific alarm settings to detect clinically relevant events^73^. Given the range of devices and alarms in typical ICUs, a human, organisational and technical factors approach to this noise source should take into account input from clinicans, manufacturers and regulators^77^.

### Strengths and limitations

This study was conducted within a bay of six patients in an adult ICU, which may limit the generalisability of the findings to other ICU departments and to ICUs with single patient rooms. The study relied on retrospective data collection from patient records. This introduces limitations predominantly due to inaccuracies in documentation. Variability in accuracy and completeness of ICU observation charts, with selective recording of abnormal observations and retrospective documentation of noisy events, when clinical workload allows, combined with subjective interpretation during analysis, may all have impacted the retrospective identification of potential noisy events.

Notably, this study assessed noise levels with national and international noise and occupational health standards for healthcare and used a site-specific Class 1 sound level meter, recording over a two-week period, representing a reliable real-world noise of care recording in an intensive care setting compared to standards seen elsewhere in the existing literature.

### Recommendations for research

We recommend that future studies consider conducting prospective analysis of sound levels in multiple centres, to increase the likelihood of reliably identifiying noisy events amongst a larger sample size. This could provide more generalisable data regarding sound levels and noise sources in ICU settings, so that interventions can be developed and tested. These interventions should be codesigned with patients and professionals. Issues with causal inference and confounding for sources of noise may be better addressed by a mixed methods approach for a national audit.

### Recommendations for clinical practice

The development and implementation of comprehensive noise reduction protocols could help to reduce excessive noise levels. These protocols should address specific sources of noise, such as alarm settings and staff communication. For instance, the introduction of smarter alarm algorithms that offer intuitive alerting to mitigate the escalation of overall noise levels^78–80^. Additionally, educating healthcare providers on the importance of noise reduction and providing human factors training on techniques to minimise noise can contribute to a quieter environment^81^. However, it is important to recognise that certain interventions may not exhibit persistent impacts or longevity, particularly in environments characterised by high staff turnover^81,82^. To address this, architectural or engineering solutions are recommended alongside behavioural strategies. Noise issues can be controlled by changing the source and transmission path between the source and receiver. Most engineering methods focus on the transmission path in the built environment. For instance, finishing materials can be replaced with sound absorption materials. Previous studies^83,84^ reported that highly absorbent ceilings are effective in reducing sound pressure levels in patient wards. Additionally, acoustic curtains or temporary enclosures can be installed during medical treatment, including like-for-like replacement of existing low-performance privacy curtains with collapsible, hydrophobic, washable, and opaque PVC coated polyester curtains, either permamently or during the noisier episodes of care this study described^85^. In a recent meta-analysis only 14 out of 25 studies demonstrated a statistically significant reduction (*p* < 0.05) in mean sound levels following an intervention^81^. These reductions were seen in almost all studies involving staff education, noise warning devices, or architectural changes^81^.

By incorporating the principles of human factors into the design process, these solutions could offer the potential for more lasting changes by reducing dependence on individual compliance^81^. We recommend exploration of design engineering approaches in co-production with professionals and patients in addition to behavioural noise management strategies to facilitate ongoing monitoring and noise reduction efforts in the ICU.

## Conclusions

This study provides valuable insight into the noise levels and source in a multi-bed adult ICU bay. Sound measurements recorded during the study period exceeded recommended parameters in international guidance. Further research is warranted to develop evidence-based interventions that promote a quieter and more conducive environment for patient care in the ICU.

## Electronic supplementary material

Below is the link to the electronic supplementary material.

Supplementary Material 1

Supplementary Material 2

## Data Availability

The datasets of sound pressure levels used and/or analysed during the current study are available from the corresponding author on reasonable request.

## Glossary

### Abbreviations

Acoustic Environment: All the sound and sound-propagating qualities of a physical environment
APACHE II: Acute physiology and chronic health evaluation II
dBA: A-weighted sound level
ICU: Intensive care unit
INC: International noise council
L_Aeq_: Equivalent continuous sound level (average sound level over a specified time period)
L_Amax_: Maximum sound level (over a specified time period)
L_Amin_: Minimum sound level (over a specified time period)
Noise: Noise suggests an element of irritation and/or harm related to sound, with many contributing factors, including psychological status, sound frequency, sound level, and context
RLUH: Royal liverpool university hospital
Soundscape: The acoustic environment as perceived, understood and/or experienced by people
USEPA: United States environmental protection agency
WHO: World health organisation
