# Supplementary material for: Noise and sound in the intensive care unit: a cohort study
Source: Sci Rep. 2025 Mar 29;15:10858. doi: 10.1038/s41598-025-94365-8 (PMC11955003; doi:10.1038/s41598-025-94365-8)

**Supplementary Appendix A**


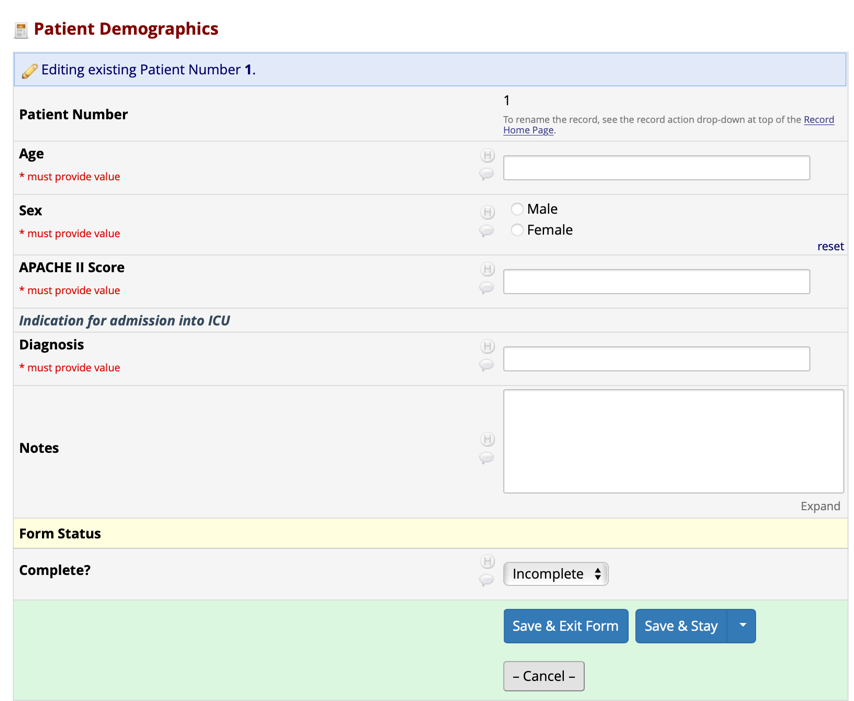


Figure 1. REDCap form A used to facilitate the study data collection for patient demographic data


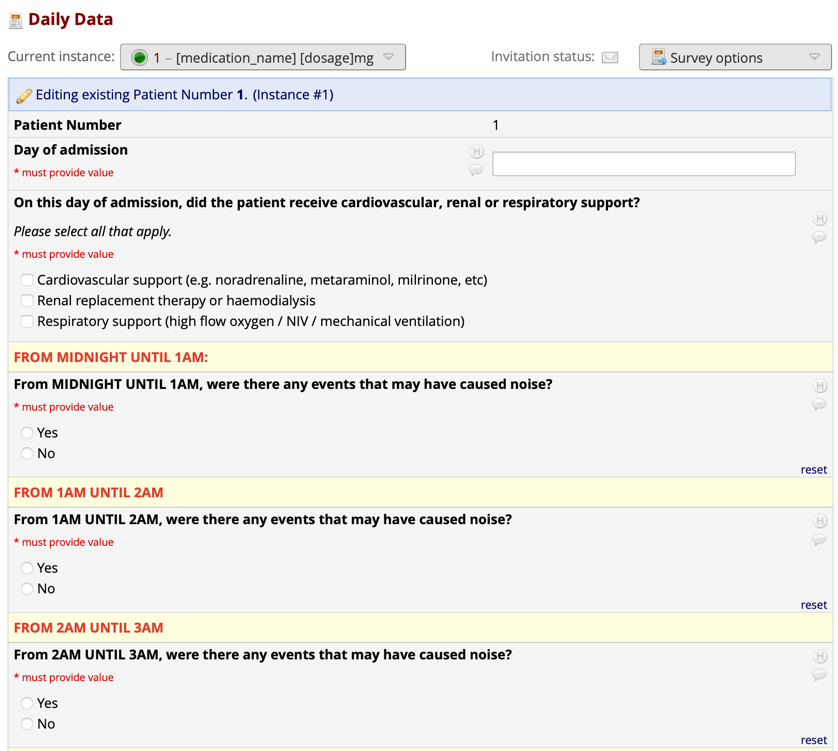


Figure 2. REDCap form B used to facilitate data collection of 'daily data' for potential noisy events

Figure 3. Exemplar REDCap form in an instance where potential noisy events have been identified


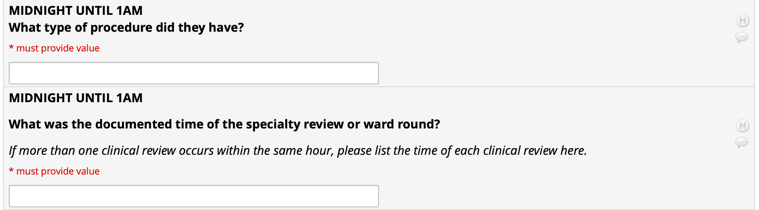

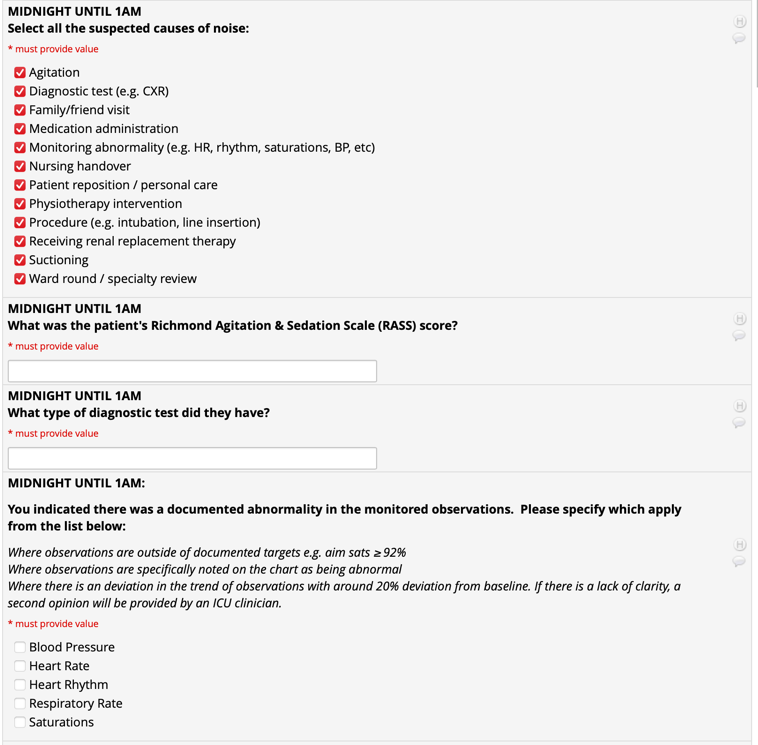

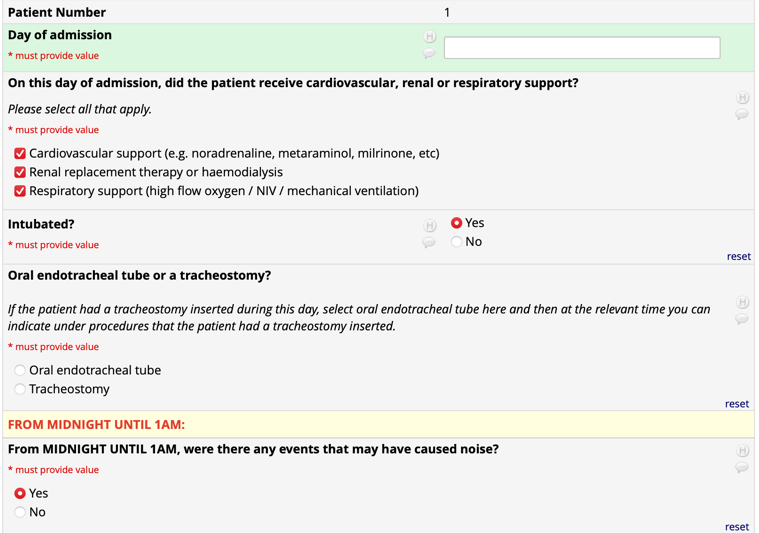

Supplement: Supplementary file 1 — Supplementary Material 1 [file 41598_2025_94365_MOESM1_ESM.docx]
